# 

**Published:** 1896-05

**Authors:** 


### EDITORIAL.

---

The office of the Business Manager of THE JOURNAL is Nos. 311 and 312 Fitten Building.

The editors are not responsible for views expressed by contributors.

Articles for publication should reach this office not later than the 15th inst.

Address all communications, and make all remittances payable to THE ATLANTA MEDICAL AND SURGICAL JOURNAL, Atlanta, Ga.

Reprints of articles will be furnished, when desired, at a nominal cost. Requests for the same should always be made *on the manuscript.*

---

### THE AMERICAN MEDICAL ASSOCIATION.

The forty-seventh annual session of this Association will be held in Atlanta from the 4th to the 8th of May. The Association met here seventeen years ago, in its thirtieth annual session. The lapse of time has wrought many changes both in the Association and in the city which entertained it.

Dr. Theophilus Parvin, then of Indiana, was President of the Association at that meeting. Among the distinguished visitors then present were Dr. S. D. Gross, of Philadelphia; Dr. N. S. Davis, of Chicago; Dr. Jno. S. Billings, U. S. Army; Dr. Stanford E. Chaille, of New Orleans; Dr. H. Knapp, of New York; Dr. Moses Gunn, of Chicago; Dr. A. C. Post, of New York; Dr. J. M. Toner, of Washington City; Dr. W. O. Baldwin, of Montgomery; Dr. Robert Battey, of Rome; Dr. Lewis A. Sayre, of New York; Dr. J. J. Woodward, U. S. Army; General Francis A. Walker, of New York; Dr. T. G. Richardson, of New Orleans, and others. The Address of Welcome, on behalf of the Committee of Arrangements, was delivered by Dr. Jos. P. Logan, who extended a "thrice-reiterated welcome to our State, our city, our homes, and our hearts."

The report of Dr. Seguin, of New York, on the adoption of the metric system was received and entered on the minutes. Dr. N. S. Davis read a report of the Committee on Ozone, and suggested the advisability of systematic daily observations concerning the "electric and ozonic conditions of the atmosphere," with respect to the "causes, conditions of spread, and consequent means for prevention of all the more important diseases." The Committee on Prize Essays reported the first prize awarded to Dr. Allen McLane Hamilton, of New York, for an essay upon "Certain Forms of Degeneration of the Lateral Columns of the Spinal Cord, with Special Reference to a Rare Infantile Form." Dr. Lewis A. Sayre, of New York, was elected President for the following year, and the present President, Dr. Beverly Cole, was made first Vice-President. The Address on Medicine was delivered by Dr. Thomas F. Rochester, of Buffalo; the Address on State Medicine and Hygiene was delivered by Dr. Billings; the Address on Obstetrics was delivered by Dr. E. S. Lewis, of New Orleans; Dr. Gunn, of Chicago, delivered the Address on Surgery and Anatomy; Dr. Knapp made a report on the Progress of Ophthalmology and Otology. These were all the sections of the Association at that time. Among other interesting papers read at that meeting was one by Dr. Sayre on the "Superior Value of the Treatment of Pott's Disease by Suspension and Retention by the Plaster-of-Paris Bandage"; one by Dr. H. F. Campbell, of Georgia, on "Urinary Calculus"; one on "State Medicine and State Medical Societies," by Dr. S. E. Chaille, of New Orleans; one on "Diseases of the Mastoid Cells," by Dr. Knapp, of New York; one on "Reparative Operations for Burns," by Dr. A. C. Post, of New York, etc.

A reception by Governor Colquitt at the Executive mansion, a visit to Stone Mountain and other interesting points in and near the city, and a barbecue at Augusta, composed the main social features of the meeting.

The Association was given a hearty welcome then, just as it is now. Atlanta then numbered forty thousand souls; to-day we have more than one hundred thousand, and as some one has already said that as Atlanta has reached its present dimensions without apparent cause, so there is equally good reasons for believing that it will yet be as large as London. We trust that this meeting of the Association will be successful throughout all the departments of its great work, in everything that will advance the glory of American medicine and surgery; also, that our visiting friends may take home with them only pleasant recollections of this meeting, of our city and of her people, and that all may feel that "it is well for us to have been there."

And when you and your successors come again, gentlemen, we will show you a bigger city, possibly more doctors, but you will not get a warmer welcome.

*We positively cannot send premium to any subscriber who does not pay a year in advance.*

NOTICE.—Our offer of a thermometer to *all* cash-in-advance subscribers is withdrawn. *New* subscribers, paying full year in advance, may still get the thermometer. Book offer still open to all who pay a year in advance. "Antisepsis and Antiseptics," "Stories of a Country Doctor."

